# The accuracy of magnetic resonance imaging in predicting the size of pure ductal carcinoma in situ: a systematic review and meta-analysis

**DOI:** 10.1038/s41523-022-00441-x

**Published:** 2022-06-29

**Authors:** Ricardo Roque, Mariana Robalo Cordeiro, Mónica Armas, Francisco Caramelo, Filipe Caseiro-Alves, Margarida Figueiredo-Dias

**Affiliations:** 1 Medical Oncology Department, Portuguese Institute of Oncology of Coimbra, Coimbra, Portugal; 2 Gynaecology Department, Hospital University Centre of Coimbra, Coimbra, Portugal; 3 Faculty of Medicine, University of Coimbra, Coimbra, Portugal; 4 Gynaecology University Clinic, Faculty of Medicine, University of Coimbra, Coimbra, Portugal; 5 Radiology Department, SESARAM EPE, Funchal, Portugal; 6 Radiology Department, Hospital University Centre of Coimbra, Coimbra, Portugal

**Keywords:** Cancer imaging, Surgical oncology, Breast cancer

## Abstract

Ductal carcinoma in situ (DCIS) is a putative precursor of invasive breast cancer and MRI is considered the most sensitive imaging technique for its detection. This study aims to evaluate the accuracy of MRI measuring the pure DCIS size, against pathology, to better understand the role of MRI in the management of this intraductal neoplasm.Potentially eligible studies in MEDLINE, Embase and Google Scholar, up to January 2021 were considered, and a systematic review and meta-analysis according to the published protocol (Prospero-CRD42021232228) was performed. Outcomes of mean differences and accuracy rates were analysed using IBM^®^ SPSS^®^ v26 and random-effect models in platform R v3.3.Twenty-two cross-sectional studies were selected and 15 proceeded to meta-analysis. MRI accurately predicted 55% of the tumours’ sizes and, according to Bland–Altman plots, concordance between MRI and pathology was greater for smaller tumours. In the meta-analysis, difference of the means between MRI and pathology was 3.85 mm (CI 95% [−0.92;8.60]) with considerable heterogeneity (I2 = 96.7%). Subgroup analysis showed similar results for sizes between different MRI fields, temporal resolution, slice thickness and acquisition times, but lower heterogeneity in studies using 3-T MRI (I2 = 57.2%). Results were concordant with low risk of bias studies (2.46, CI 95% [0.57–4.36]), without heterogeneity (I2 = 0%).Therefore, MRI is shown to be an accurate method in pure DCIS size assessment. Once the best MRI protocol is established, evaluation of the impact of pure DCIS size in predicting treatment outcomes will contribute to clarifying current issues related to intraductal breast carcinoma.

## Introduction

Ductal carcinoma in situ (DCIS) represents a heterogeneous group of malignant epithelial proliferations confined to the mammary ducts, constituting a known precursor of invasive carcinoma. It affects up to 20% of women, with an increasing incidence following screening mammography^1,2^.

The standard of care in DCIS therapy is surgical treatment, either breast conserving surgery (BCS) or mastectomy, which can be followed by radiotherapy. Such an invasive approach reflects the current incapacity to stratify the aggressiveness of this tumour^1^. Therefore, over diagnosing indolent DCIS and underdiagnosing more aggressive lesions is a major concern, leading to an increasing interest in novel DCIS diagnostic approaches. Preoperative accurate DCIS size evaluation is extremely important to optimize surgical planning and prevent unnecessary mastectomies. Complete tumour excision determines the success of BCS, lowering re-excision rates and its inherent comorbidities and reducing recurrence^1,2^.

Nowadays, increasing evidence suggests that magnetic resonance imaging (MRI), namely dynamic contrast enhanced MRI (DCE-MRI), is the most sensitive imaging technique available for the diagnosis of DCIS^3–6^. Nevertheless, MRI assessment of tumour size is still challenging, because of the dominant presentation of DCIS as a non-mass enhancement (NME) lesion. Regardless of the cumulative evidence on this topic, the impact of MRI in determining the size of the DCIS prior to surgery and its accurate correlation to pathology measurements remains controversial^2,5^. To our knowledge and to date, there is no extensive review work on this subject. Therefore, our systematic review and meta-analysis aimed to assess the diagnostic accuracy of MRI in measuring the histopathology-proven pure DCIS tumour size and its protocol’s demands, to better understand the role of MRI in the clinical management of this breast neoplasm.

## Results

### Selected studies characteristics

**Fig. 1:**
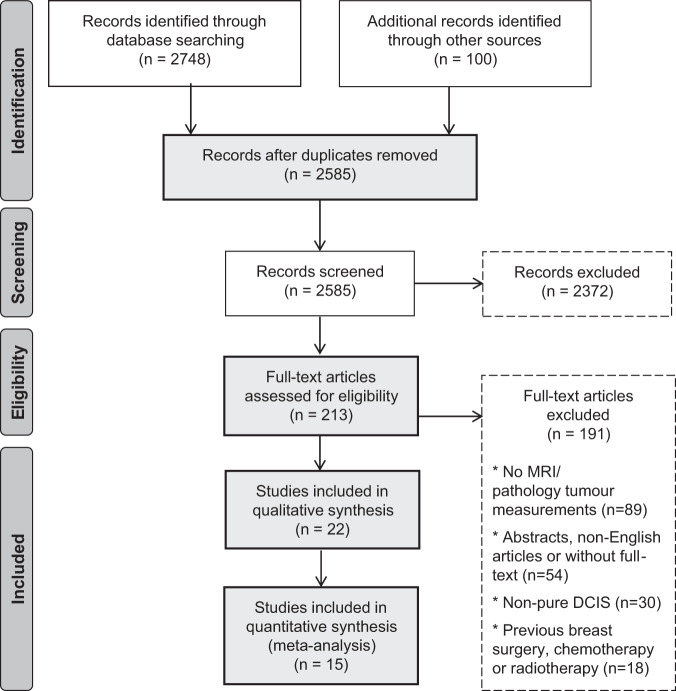
Flow diagram of studies selection methodology, according to PRISMA flow chart^9^. References were selected from the databases applying the search equation and then selected by relevance of title and abstract. Final selection relied in the application of inclusion and exclusion criteria.

Article selection followed the Prisma flow chart shown in Fig. 1^7–9^. From the 2585 records identified, only 8.2% were selected for full-text reading. Abstracts and titles regarding other breast and non-breast tumours, or not showing MRI or pathology examination were the main causes for exclusion at this phase. From the 213 records selected for full text assessment, 89 did not have tumour quantitative size measurements in MRI and/or histopathology; 54 were abstracts, non-English articles, or the full text could not be found; 30 concerned non-pure DCIS; and 18 included women with previous breast surgery, chemotherapy, or radiotherapy. Many of the records rejected matched more than one criterion.

The year of publication ranged from 2005 to 2020. Seven studies were performed in Asia (five of which were in South Korea), seven in Europe (Switzerland, Netherlands, France, the UK, and three in Germany), seven in the USA and one in Australia. All articles were cross-sectional diagnostic accuracy studies and used contrast enhanced MRI (DCE-MRI), except for two studies that did not present any information regarding MRI protocol specifications (Kumar et al., 2006; Vanderwalde et al., 2010).

More than 1300 patients where included, with a mean age of 52 years, and a total of 1247 DCIS lesions measured by MRI, with 60.85% of the tumours revealing a prevalent pattern of non-mass enhancement (NME). Relevant information regarding the population and MRI specifications of the 22 included studies is presented in Table 1.

**Table 1:** Characteristics of included studies.

Study		Menell	Esserman	Kumar	Ornesti	Marcotte-Bloch	Leung	Allen	Vander- walde	Shin	Baur	Mun	Gruber	Pickles	Rahbar^a^	Rominger	Baek	Daniel	Brennan	Preibsch	Song	Sanderink	Shiraishi	Total/ Mean
Study publication year		2005	2006	2006	2008	2009	2010	2010	2010	2012	2013	2013	2013	2015	2015	2015	2017	2017	2017	2019	2020	2020	2020	NA
DCIS patients (*N*)		32	45	45	16	33	9	63	11	88	58	212	15	26	20	10	56	225	17	123	50	46	164	1364
DCIS measured in MRI (*N*)		34	45	45	16	32	9	64	9	88	58	78	15	26	19	10	55	244	17	123	50	46	164	1247
DCIS patients mean age [years (SD)]		53.0	50.0	–	–	59.7 (10.3)	47.5 (8.4)	61.9	–	–	52.0	50.0	–	–	54.9	–	51.0	52.4 (10.3)	–	55.5	–	–	54.0	53.2^b^ (3.2)
Biopsy proven DCIS		Yes	Yes	Yes	Yes	Yes	No	Yes	Yes	No	Some	Yes	No	Yes	Yes	Yes	Some	Yes	Yes	Some	Yes	Yes	NA	NA
MRI field [*N* (%)]	1.5 T	✓	✓		✓	✓	✓	✓		✓	✓	✓	✓			✓	✓		✓	✓			✓	15 (68)
	3 T													✓	✓			✓			✓			4 (18)
	NA			✓					✓													✓		3 (14)
Contrast type [*N* (%)]	Gadolinium-based	✓	✓			✓	✓	✓		✓	✓	✓	✓	✓	✓	✓	✓	✓	✓	✓	✓		✓	18 (82)
	Gadopentetate^c^	✓						✓			✓	✓				✓	✓						✓	7 (32)
	NA			✓	✓				✓													✓		4 (18)
Contrast dose (mmol/kg)		0.10	0.10	–	–	0.10	0.20	0.10	–	0.10	0.16	0.20	0.16	0.05	0.10	0.20	0.10	0.10	0.10	0.10	–	–	0.10	0.17^d^ (0.05)
Contrast flow rate (ml/s)		–	1.0	–	–	–	–	3.0		–	–	1.0	–	–	2.0	2.0	1.0	2.0	2.5	–	2.0	–	1.0	1.75^d^ (0.23)
MRI captures after contrast (*N*)		–	2	–	–	3	–	–	–	4	7	5	7	7	3	9	5	6	4	7	5	–	3	5^d^(2)
DCE-MRI scan time^e^ (s)		–	300	–	–	420	–	480	–	–	525	305	–	420	480	550	300	–	–	525	300	–	240	400^d^ (29)
Temporal resolution^e^ (s)		–	–	–	–	–	90	–	–	–	75	61	–	60	–	55	60	–	90	75	60	–	–	70^d^ (4)
Slice thickness^e^ (mm)		2.0	2.0	–	–	1.4	1.1	–	–	1.0	1.5	0.9	–	^f^	1.3	2.0	0.9	3.0	–	1.5	2.0	–	–	1.5^d^ (0.2)
Matrix size (mm)		259 × 192	–	–	–	256 × 256	512 × 512	–	–	224 × 448	512 × 512	–	–	^f^	440 × 660	512 × 512	384 × 384	280 × 512	–	512 × 512	320 × 320	–	768 × 768	NA
Field of view (cm)^g^		18 × 18	–	–	–	–	36×36	–	–	17 × 17	40 × 40	34 × 34		^f^	22 × 32	38 × 38	34 × 34	32 × 32	–	40 × 40	32 × 32	–	34 × 34	NA
Patient position		Prone	–	–	Prone	Prone	Prone	Prone	–	–	–	–	–	–	–	Prone	Prone	Prone	–	Prone	Prone	Prone	Prone	NA
Breast coils		Yes	–	–	Yes	Yes	Yes	Yes	–	Yes	Yes	Yes	–	Yes	Yes	Yes	Yes	Yes	Yes	Yes	Yes	Yes	Yes	NA
Channels/breast coil (*N*)		–	–	–	–	–	–	8	–	–	–	4	–	8	16	–	–	8	8	7	–	–	–	8^d^ (3)
DCIS Enhancement	mass (%)	34	–	–	–	22	–	–	–	–	46	65	–	–	30	30	60	–	–	–	50	–	20	40^b^ (18)
	Non-mass (%)	66	–	–	–	78	–	–	–	–	54	35	–	–	70	70	40	–	–	–	50	–	80	61^b^ (18)

NA non-applicable and “–” non-available.

^a^In Rahbar 2015 two different sets of measurements were made using 1.5 and 3 T MRI. Only results for 3 T MRI are presented.

^b^Weighted mean and SD in brackets.

^c^Gadopentetate dimeglumine is a type of Gadolinium-based contrast. It was the only specified contrast that appeared in the included studies.

^d^Simple mean and SD in brackets.

^e^These parameters refer to the DCE-MRI protocol.

^f^This study applies multiple DCE-MRI settings in one protocol, presenting different slice thickness and matrix sizes.

^g^For Menell et al. and Leung et al. only the largest fields of view are presented in the table. The abtracted range is 16 × 16 to 18 × 18 for Menell et al. and 20 × 20 to 36 × 36 for Leung et al.

### DCE-MRI protocol specifications

All 18 studies that present information regarding the type of contrast applied, used a Gadolinium-based contrast, specified in 7 articles as Gadopentetate dimeglumine. The majority of studies used a contrast dose of 0.1 mmol/kg and some used a contrast dose that varied between 0.05 and 0.2 mmol/kg. Among the 22 included studies, a total of 18 mentioned the use of a dedicated breast coil, but only 7 mentioned the number of channels used, which ranged between 4 and 16 channels. Regarding the spatial resolution, slice thickness varied from 0.9 to 3.0 mm as shown in Table 1.

Total DCE-MRI scan time protocol from the beginning of IV contrast injection to the final image acquisition varied between 300 and 550 s, and temporal resolution, or time between each image captured after contrast injection, varied between 60 and 90 s.

In the included studies, axial and/or sagital planes were used for tumour measurement and all of the studies used a bidimensional maximum tumour measurement for both MRI and pathology. Only a few studies specified in which MRI plane and in which DCE acquisition time the tumour measurement was performed. Because these variables were chosen individually for each tumour by the radiologists in most studies, this information was not analysed in our systematic review.

### Reported tumour sizes and accuracy

Reported information regarding the measurements of tumour size and the diagnostic accuracy of the MRI is presented in Table 2. According to Rahbar et al., 2015, the accuracy of 3.0 and 1.5 T MRI is compared. Therefore, we chose to present the results of the 3.0 T MRI, because they demonstrated a better correlation to pathology in this study and it also allowed for further correlation with other 3.0 T MRI results from other articles^10^. In Shiraishi et al., 2020, a comparison of MRI evaluation between two different protocols (abbreviated and full diagnostic) was made. In this case, we chose to incorporate information from the full diagnostic protocol, that showed a better correlation between the MRI and pathology^11^.

**Table 2:** Abstracted tumour size and accuracy of MRI measurements.

Study		Menell	Esserman	Kumar	Ornesti	Marcotte-Bloch	Leung	Allen	Vander- walde	Shin	Baur	Mun	Gruber	Pickles	Rahbar^a^	Rominger	Baek	Daniel	Brennan	Preibsch	Song	Sanderink	Shiraishi	Total/ Mean
Study publication year		2005	2006	2006	2008	2009	2010	2010	2010	2012	2013	2013	2013	2015	2015	2015	2017	2017	2017	2019	2020	2020	2020	NA
DCIS measured in MRI (*N*)		34	45	45	16	32	9	64	9	88	58	78	15	26	19	10	55	244	16	123	50	46	164	1247
Mean MRI size [mm (SD)]		–	–	47.6	31.1 (4.4)	28.1 (20.8)	27.4 (21.2)	–	6.4 (8.8)	31.7 (21.3)	43.6	–	–	49.6 (26.8)	18.2	–	23 (17)	25.3 (18.4)	42.3 (19.0)	–	25.8 (15.5)	19.2 (10.5)	–	29.6^b^ (9.1)
Mean pathological size [mm (SD)]		–	–	32.4	18.2 (4.9)	25.6 (20.0)	18.0 (11.1)	–	28.3 (28.8)	30.5 (20.1)	39.1	–	–	50.6 (34.2)	14.1	–	20 (18)	18.8 (13.6)	58.2 (35.0)	39.6	25.0 (18.0)	21.8 (17.5)	38	24.5^b^ (10.0)
Accuracy	Overestimation (%)	–	23.0	–	50.0	21.0	–	46.5	–	–	42.3	–	–	–	–	30.0	18.2	29.5	37.5	–	32.0	26.1	12.0	27.3^b^ (10.8)
	Accurate (%)	–	68.0	–	50.0	60.0	–	34.9	–	–	26.9	–	–	–	–	50.0	72.7	61.1	56.3	–	36.0	47.8	61.5	54.9^b^ (13.0)
	Underestimation (%)	–	9.0	–	0.0	19.0	–	18.6	–	–	30.8	–	–	–	–	20.0	9.1	9.4	6.2	–	32.0	26.1	26.5	17.8^b^ (9.2)
Margin of error (mm)^c^		–	–	–	5	5	–	1	–	–	10	–	–	–	–	10	10	10	20	–	5	5	10	NA
Correlation coefficient		–	0.55	–	–	0.83	–	–	–	–	0.74	–	0.74	–	0.66	–	0.74	0.41	0.42	–	0.62	–	0.69	NA

*NA* non-applicable, “–” non-available.

^a^In Rahbar 2015 two different sets of measurements were made using 1.5 and 3 T MRI. Only results for 3 T MRI are presented.

^b^Weighted mean and SD in brackets.

^c^Each study stablished a margin of error to define the maximum size difference accepted to define concordance between MRI and Pathology.

Numeric data related to mean difference and LOA was only found in 4 articles (Gruber et al., 2013; Pickles et al., 2015; Rahbar et al., 2015; Daniel et al., 2017), and one of these (Pickles et al., 2015) presented data after logarithmic transformation^10,12–14^. Two other articles (Rominger et al., 2016; Sanderink et al., 2020) reported Bland–Altman analyses only with a graphic representation^15,16^. Comparison of the Bland–Altman plots showed a similar dispersion of the measurements, with greater concordance between MRI and pathology for tumours of smaller sizes. As the DCIS becomes larger, discordance between measurements increases progressively, without a clear tendency for over or underestimation^10,12–16^. Different plots are shown by Rahbar et al. (2015) and Pickles et al. (2015), which used a 3.0 T field, instead of 1.5 T. In these plots, less dispersion is shown, with more consistent results regarding concordance of measurements for all tumour sizes^10,13^. Rahbar et al. (2015), also shows a narrower 95% LOA for a 3.0 T MRI, when compared to a 1.5 T, for the 19 tumours measured^10^.

**Fig. 2:**
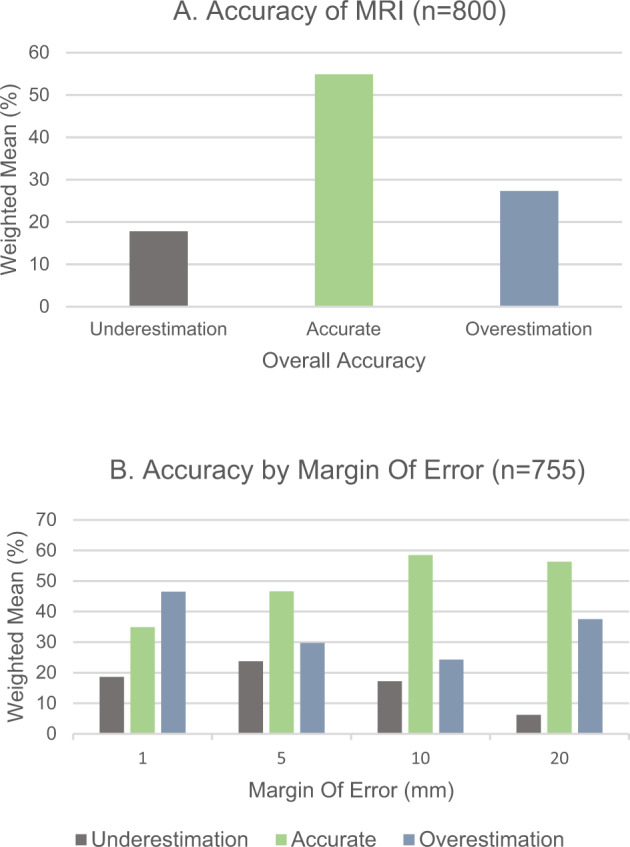
Percentage of concordance between MRI and pathology. Weighted mean percentage of concordance, overestimation and underestimation between MRI and pathology measurements in the 12 studies with different predefined margin of error, according to Table 2 (**A**). Weighted mean percentage of concordance, overestimation and underestimation between MRI and pathology measurements in 11 studies with equal predefined margin of error (**B**).

Of all the articles included, presented in Table 1, 13 presented accuracy percentages, but only 12 of these established a margin of error. A graphic summary of these results is presented in Fig. 2. With margins of error of 5 mm or more, with the exception of one study (Baur et al., 2013)^17^, we found there was a higher percentage of accurate measurements of tumour size by MRI. In pooled concordant results, 54.9% of the tumours were accurately measured by MRI, followed by a tendency for overestimation.

### Meta-analytic results of MRI accuracy in pathologic size estimation

The low number of Bland–Altman analyses led us to use the difference between the means of tumour size for our meta-analysis. A total of 15 studies included in the final analysis had random sets of data missing (Little, *p* = 0.434). Eight values for each SD and the correlation coefficient were obtained by imputation. The imputed values can be found in the Supplementary Table 1.

**Fig. 3:**
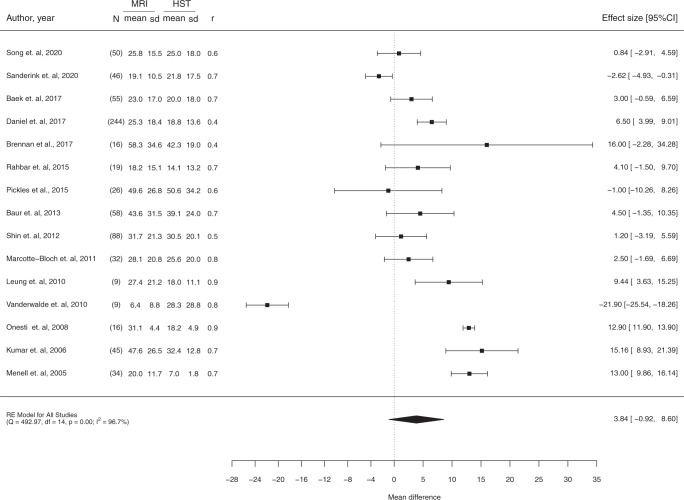
Mean size difference between MRI and pathology across studies. Pooled results of the mean size difference of paired measurements of DCIS with MRI and Pathology (HIST), using an imputation method, for all the eligible studies.

When considering imputed values, the difference of the means between MRI (MRI) and Histopathology (HIST) was 3.85 mm (CI 95% [−0.92; 8.60]). According to the forest plot in Fig. 3, there is no statistical difference between the size of DCIS evaluated with MRI and pathology. According to Cochrane’s handbook, the results present considerable heterogeneity (I2 = 96.7%; Cochran’s *Q* *p* < 0.00)^18^.

### Heterogeneity and bias assessment

**Fig. 4:**
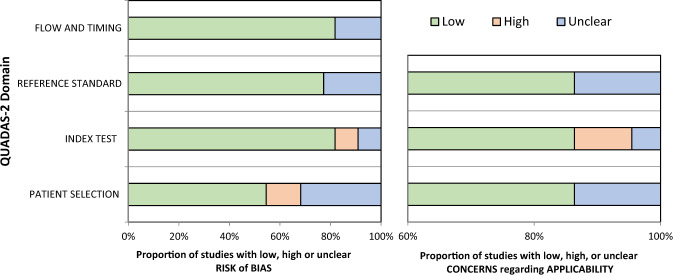
Risk of bias evaluation according to QUADAS-2 tool. Adapted from the template for graphical display at https://www.bristol.ac.uk/population-health-sciences/projects/quadas/resources/.

Regarding the risk of bias, the results from the QUADAS-2 evaluation are presented in Fig. 4 and the corresponding table of risk assessment can be found in Supplementary Table 2^19^. It shows an almost 50% rate of high or unclear risk of bias regarding the patient selection factor. In combination with other evaluated factors, this translates into a result of 13 out of 22 of the studies we included have a risk of bias. In the applicability section, high concerns were raised for the MRI measurements in 10.5% of the studies^19^.

**Fig. 5:**
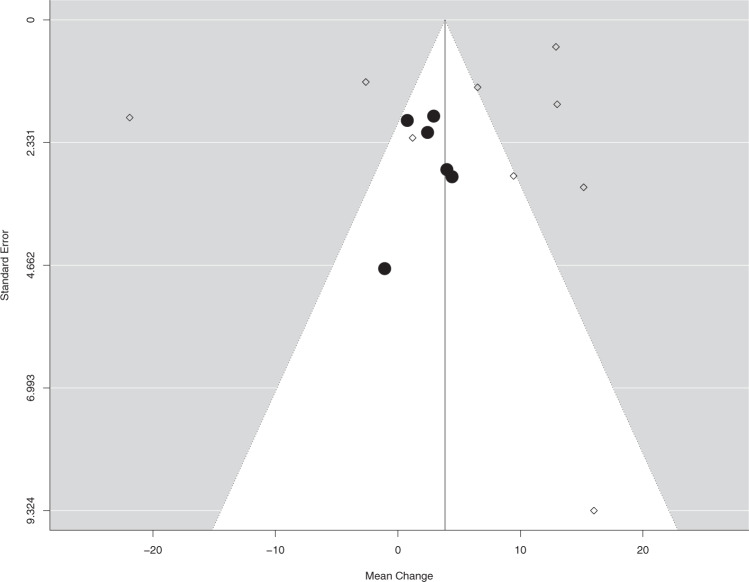
Publication bias assessment with a funnel plot. In the presented graphic dispersion, each study is marked according to its risk of bias, evaluated according to QUADAS-2 tool. Studies with risk of bias are represented by diamonds () and without risk of bias by circles ().

To assess publication bias, we conducted a funnel plot, as presented in Fig. 5, highlighting the studies with risk of bias according to QUADAS-2 (diamonds). Visual evaluation of the plot indicates there is no evident asymmetry^18,19^.

A sensitivity analysis was performed to investigate the sources of heterogeneity. Even though all corresponding authors were contacted, we were unable to obtain more hard data. Concerning imputation methods, a model with no imputed values comprising only 5 articles (versus 15 articles in the imputed model), resulted in statistically significant differences in the overall effect size (4.97, CI 95% [1.48; 8.47]), but when compared with the imputed results the confidence intervals overlap, maintaining substantial heterogeneity (I2 = 64.6%). Considering the six studies with a low risk of bias according to QUADAS-2, the confidence intervals of the summary measure overlap showing no difference in overall effect (2.46, CI 95% [0.57; 4.36]) and no heterogeneity was detected (I2 = 0%, Cochran’s *Q* *p* = 0.83)^18^.

In subgroup analysis, no statistically significant differences in overall effect size were observed between studies using different MRI fields (3.47, CI 95% [0.02, 6.92] for 3.0 T versus 7.18, CI 95% [3.34, 11.01] for 1.5 T), acquisition times (1.97, CI 95% [−0.63, 4.56] for <300 s versus 3.03, CI 95% [0.25, 5.81] for >300 s), time resolution (−1.89, CI 95% [−4.67, 0.89] for 60 s or less versus −8.71, CI 95% [−13.83, −3.60] for more than 60 s), or slice thickness (3.53, CI 95% [1.63; 5.43] for 1.5 mm or less versus 6.94, CI 95% [0.04; 13.63] for greater thicknesses). Moderate to substantial heterogeneity (I2 = 57.2%), was obtained in the subgroup of studies using 3 T MRI. No heterogeneity was observed for time resolution of 60 s or less (I2 = 0.0%, Cochrane’s *Q* *p* = 0.66) or slice thickness of 1.5 mm or less (I2 = 0%, Cochrane’s *Q* *p* = 0.36), in opposition to a moderate to substantial heterogeneity observed for time resolution of more than 60 s (I2 = 67.2%, Cochrane’s *Q* *p* = 0.06) and considerable heterogeneity for slice thickness greater than 1.5 mm (I2 = 93.1%, Cochrane’s *Q* *p* = 0.00). No heterogeneity was detected between subgroups with different acquisition times (I2 = 0.0%, Cochrane’s *Q* *p* = 0.76 for acquisition times >0.3 s and *p* = 0.41 for acquisition times <0.3 s)^18^.

Using the last 5 years as a surrogate indicator for MRI technological advances, subgroup analyses showed no statistically significant differences in overall results (2.35, CI 95% [−0.77, 5.47]), with the interception of the line of no effect^18^. Forest plots for subgroup and sensitivity analyses can be found in the supplementary information (Supplementary Figs. 1–11).

## Discussion

Increasing interest in MRI accuracy in preoperative DCIS size measurement translates into a need for a consensus about the value of this imagiological technique in non-invasive breast neoplasm evaluation.

According to the concordance between studies included, MRI accurately predicts tumour size in the majority of the cases, within a predefined margin of error^11,14,15,20–23^, whereas in the remaining DCIS cases, a tendency for overestimation was revealed by most studies. For a margin of error >10 mm, higher accurate size measurements were observed and our results suggest that a 10 mm cut-off is associated with the greatest concordance rate for MRI-pathology^11,14,15,17,22,23^. Despite the high heterogeneity and concerns related to the risk of bias in some of the included studies, the meta-analytical results presented in Fig. 3 confirm these findings with moderate certainty, along with a mean difference between pathology and MRI measurements of 3.85 mm. Regardless of the low number of Bland–Altman analyses in the selected studies, concordance between MRI and pathology is particularly evident in small size tumours^10,12–16^ and increases with higher MRI field strength^10,13^.

In the meta-analysis, Vanderwalde et al. represents an obvious outlier which can be attributed to problems of methodology and low number of DCIS included. Its MRI protocol and equipment specifications are not reported, and it has raised serious concerns about its index test and its applicability in QUADAS-2 evaluation. Brennan et al., stands out for its wide range of overestimation and this discordance is expected, due to a large mean DCIS size in a small study sample. Likewise, Menell et al., Kumar et al., and Ornesti et al., are small and older studies, where there might be concerns about the poor quality of MRI evaluation and the risk of bias, according to QUADAS-2 evaluation, mainly for Kumar et. al.

The impact that different MRI protocols have on the size measurements of DCIS is of extreme clinical interest. In fact, in DCE-MRI, most breast tumours show peak enhancement at ~60–90 s after contrast injection. Therefore, by convention, a time resolution of ~90 s is used, and captures beyond that time will improve differentiation between breast lesions^3^. Our results show that later DCE-MRI readings do not perform better at DCIS size estimation, because longer exams (over 300 s post-contrast injection) did not predict DCIS size with any better accuracy and even presented a larger CI for size difference^11,22^. However, longer scan times may be of interest when accurately diagnosing DCIS using MRI for differential diagnosis with other breast lesions^3^. On the other hand, lower time resolutions are related to better results when predicting tumour size, despite the absence of statistical significance.

The diagnostic performance of breast MRI can also be influenced by the contrast type used, and in recent evidence, Gadopentetate Dimeglumine has been recognized as a preferable contrast compound^24,25^. Nevertheless, most of the studies did not report the type of contrast used, so differences in Gadolinium contrast agents could not be evaluated. Contrast material should be dosed at of 0.1 mmol/kg according to state of the art recommendations^3^, and in low risk of bias studies, only Pickles et al. used an inferior dose. The impact of this in its results is not possible to predict, however it is the unbiased study with the wider confidence interval.

Regarding magnetic field strength, 3.0 T MRI allows for better contrast, and spatial and temporal resolution^10,21^ and according to Rahbar et al., 2015, leading to a more accurate assessment of DCIS, compared to 1.5 T^10^. Our results showed higher accuracy for tumour size measurements in the 3.0 T MRI with a lower CI, even though not statistically significant.

Increasing spatial resolution is also of the utmost importance for evaluating tumour morphology and contributing for breast lesions differential diagnosis particularly considering non-mass lesions. A slice thickness lower than 3 mm, which is the standard thickness used based on the American College of Radiology (ACR) breast MRI accreditation standards, was uniformly respected among included studies^3^. Despite the non-statistical significance, analysis of our subgroups indicated the positive impact of reducing slice thickness and increasing spatial resolution for the accurate measurement of DCIS.

Breast coils are also mandatory in diagnostic breast MRI, and this condition was uniformly fulfilled by all studies. At least four channels are required, but Rahabar et al. with outcomes coherent with our overall meta-analytic results, used 16 channels as in more updated breast coil designs^3^.

Meta-analytic results did not explain the MRI tendency for overestimation, due to lack of available information. Multiple external factors, which were not evaluated in our work, may influence MRI capacity for tumour size assessment. As previously shown^26^, our pooled results exhibit a tendency for NME as the main presentation of DCIS. Despite the lack of unanimous agreement in the literature about the relation between tumour three-dimensional distribution and MRI accuracy^17,22^, NME has been indicated as predictive of size discordance between MRI and pathology^26^.

Higher background parenchymal enhancement also contributes to DCIS size overestimation^14^, mainly in a scattered or widely distributed DCIS^27,28^. It may be caused by post-biopsy residual inflammation, increased capillary permeability^21,27–31^, nearby fibrocystic alterations, sclerosing adenosis, atypia^13,17,27–29,32^, or menstrual cycle phase^10^, with Daniel et al., 2017, showing a better agreement in size estimation with MRI for women over 50 years^14^. In almost all the included studies, MRI was performed after core needle biopsy and the presence of benign breast changes cannot be excluded, so we should consider the potential interference of these factors in our results.

Prone position in MRI stretches the breast, changing its shape, whereas pathology measurements are done in a non-prone position^22,33^. Another possible reason for a tendency for DCIS size overestimation with MRI could be attributed to size underestimation by the two-dimensional pathology analysis. Therefore, MRI’s three-dimensional analysis advantage might make it difficult to corelate to the bidimensional gold-standard^13,15,27,29^. As Rominger et al., 2015, mentioned, the orientation of the specimen slicing can also explain this phenomenon, especially when “large or non-palpable tumours are sliced along the anatomical organ axis, rather than along the main tumour axis”, as they are measured in MRI^15^. This may explain why Shiraishi et al. (2020), matching the measurement planes of MRI and pathology, show higher agreement rates. In addition, several studies denoted that formaldehyde fixation causes shrinkage in tumour specimens^11,15,17^. So, DCIS intrinsic characteristics and the different natures of MRI in vivo analysis, in comparison with pathology ex vivo methods, may contribute to this discordance and lead to the hypothesis that pathology size measurements, as a gold standard, may not fully correlate with real tumour size before surgical treatment^13,15,20,29,33^.

The high heterogeneity found in the meta-analytical results can be attributed to multiple causes. Inter-protocol variability may explain it, and, indeed, heterogeneity in the subgroup analysis was lower. To further address this issue, the risk of bias was used as a measurement for protocol quality. Accordingly, studies with a lower risk of bias showed results overlapping the general meta-analysis, but without heterogeneity^19^.

The use of an imputation method to deal with missing data may be seen as a limitation. However, only standard deviation and correlation coefficient values were imputed, and sensitivity analysis showed no statistically significant differences between pooled results before and after imputation.

Regarding publication bias, potential imbalances displayed in the funnel plot can be explained by the influence of smaller studies with serious concerns regarding methodology (high risk of bias), and larger studies that did not provide enough data for meta-analysis^18^.

MRI protocols and technology have been experiencing an impressive evolution^34^. Surprisingly, when considering studies published in the last 5 years that may have involved more contemporary MRIs, no changes in the overall effect on size were found. Therefore, the impact of this technological evolution needs to be followed by the implementation of accurate MRI protocols, to achieve the full diagnostic potential of MRI.

Despite the absence of important hard data regarding pathology, MRI methodology, and population characteristics, we were able to compare the impact of different MRI features in the DCIS measurement and discuss their potential implications in MRI analysis. Although limitations regarding missing data, the small number of included studies, and reservations related to the suitability of the established gold-standard technique, our meta-analysis reflects a clear need to determine which MRI protocol specifications are better suited for DCIS size determination.

The consensus among the Society of Surgical Oncology (SSO), the American Society for Radiation Oncology (ASTRO), and the American Society of Clinical Oncology (ASCO) is that a breast conservative approach for DCIS requires an at least a 2 mm cancer-free margin^35^. Previous systematic reviews state that preoperative MRI does not improve surgical management of DCIS patients, namely the rates of mastectomy, positive margins, or reintervention^33,36^. However, these studies ignore more recent research and the impact of the DCE-MRI in the final outcomes^33,36^. Despite the absence of statistically significant results in our protocols subgroup analysis regarding the cancer free margin threshold, slight differences in size estimation found when comparing DCE-MRI protocols may have a clinical impact^4,35^.

For future directions, the creation of a refined protocol that is specific to DCIS tumour morphologic and kinetics characteristics seems to be of the utmost importance. Simultaneously, the more accurate MRI measurements observed for smaller tumour sizes highlights the potential of MRI screening for detecting DCIS at earlier stage and less aggressive behaviour.

The relevance of breast MRI in pure DCIS management is raising increasing interest^5^. The routine use of preoperative MRI in measuring DCIS tumour size is less studied compared with invasive breast cancer. Nevertheless, recent evidence suggests that MRI can possibly have a higher accuracy than mammography in assessing disease extent^5,32,33^. Taken together, the results of the present meta-analysis support the hypothesis that, despite the tendency of size overestimation by MRI, this imaging method allows for an accurate DCIS size estimation^33^.

The impact of an accurate pre-surgical DCIS size estimation on the clinical outcomes of breast cancer patients is beyond the scope of this study^33^^,36^. Future studies are recommended concerning DCIS size measurement by a refined DCIS-specific MRI protocol. The precise evaluation of pure DCIS size in predicting surgical outcomes would help solving one of the intraductal breast carcinoma’s current issues^6^.

## Methods

### Sources and search strategy

The protocol for this systematic review and meta-analysis is registered online in Prospero since 28 January 2021 with the following ID: CRD42021232228.

The search for eligible studies was made in MEDLINE, Embase and Google Scholar, from their origin until January 2021. The MeSH terms “Carcinoma intraductal non-infiltrating” and “Magnetic resonance imaging”, as well as the word “size”, and other related synonyms were used for database search according to the following search equations:

- Medline: ((Magnetic Resonance Imaging[MeSH Terms]) OR (MRI[Title/Abstract]) OR (magnetic resonance[Title/Abstract])) AND ((DCIS[Title/Abstract]) OR (Carcinoma, Intraductal, Noninfiltrating [MeSH Terms]) OR (ductal carcinoma in situ[Title/Abstract])) AND (size[Text Word]).
- Embase: ((‘nuclear magnetic resonance imaging’/exp OR mri:ab,ti OR ‘magnetic resonance’:ti) AND ‘breast carcinoma in situ’/exp OR dcis:ab,ti OR ‘intraductal carcinoma’/exp OR ‘ductal carcinoma in situ’:ab,ti) AND size:ti,ab,kw.
- Google Scholar: (allintitle: MRI AND DCIS) OR (allintitle: MRI AND Ductal Carcinoma in Situ) OR (allintitle: magnetic resonance AND Ductal Carcinoma in Situ) OR (allintitle: magnetic resonance AND DCIS).

Grey literature and backward citation searches were also performed. Due to the high number of hits in Google Scholar, only titles containing the aforementioned MeSH terms or related synonyms were searched in this database.

### Studies selection

All searching records were managed using Mendeley^®^ software. Two authors independently selected potentially eligible articles based upon titles and abstracts, and disagreements were discussed between both. Then, using the same methodology, they evaluated the resulting articles through full-text assessment, according to inclusion and exclusion criteria. During this process, blinding was not applied to the review authors regarding the publication journal, studies’ authors, or institutions.

The included studies involved adult females older than 18 years, with any kind of breast type according to BIRADs classification and any risk of breast cancer, with established pathological diagnosis of one or multiple primary or recurrent pure DCIS, affecting one or both breasts, in any clinical setting. Studies with both MRI and pathology tumour analysis protocols with quantitative tumour size measurements were included. Only randomized controlled trials, cross-sectional and longitudinal studies were considered. Studies that included women with preoperative chemotherapy or radiotherapy treatments, with any microinvasion focus, articles written in non-English languages, and abstract-only publications were excluded.

### Data abstraction

The two authors used standardized forms to independently abstract the relevant information from the included articles, regarding author, publication date, study design, sample size, and patient age, MRI protocol or protocols used, number of participants per protocol, type of contrast administered and its dosage, temporal and spatial resolution, MRI planes and image types captured, mean tumour size, and information about how tumour size was defined in histopathological samples and in MRI images.

Regarding tumour size concordance between MRI and histopathological examination, we gathered the bias and 95% associated limits of agreement (LOA), following the method described by Bland and Altman^37^. Interclass, Spearman’s and Pearson’s correlations, as well as the percentage of agreement and under/overestimation within a chosen margin of error were also abstracted when present.

To obtain missing data of interest, authors of the accepted articles were contacted.

### Data analysis and synthesis

In addition to systematic narrative synthesis, a summary of the most relevant data of the articles was made with descriptive statistics, using IBM^®^ SPSS^®^ v26. Meta-analysis was also performed based on a random effects model^38^ using the metafor package for the platform R version 3.3.2^39^. Because most articles did not provide the mean difference and LOA, but only correlation coefficients, mean sizes, and standard deviation of both pathologic and MRI measurements, we considered the samples as paired and used the difference of the means for the meta-analysis. In the cases where additional information from the authors was impossible to obtain, we used an imputation method to deal with missing values of standard deviations and correlation coefficients only. MCAR missing type was evaluated through Little’s test and imputation was performed using IBM^®^ SPSS^®^ v26 with a regression-based model. All articles without mean pathological or MRI measured size were excluded from the meta-analysis. A significance level of 0.05 was adopted throughout the systematic review.

Heterogeneity was measured through inconsistency I2 and Cochran’s *Q* test (with a significance level of 0.1), according to Cochrane Handbook for Systematic Reviews of Interventions Version 6.2. Subgroup analysis was performed according to the availability of information, using MRI field intensity, DCE-MRI scan time, time resolution, slice thickness, and year of publication. In the absence of background literature, thresholds for subgroup analyses, regarding times and sizes, were established using the mean of the abstracted values eligible for meta-analysis as reference. To assess the interference of the risk of bias and imputation methods in the meta-analytic results, we used a sensitivity analysis and a funnel plot^18^.

### Studies and review quality assessment

Despite the protocoled strategies for risk of bias assessment, and because all accepted references were cross-sectional diagnostic accuracy studies, we used the QUADAS-2 tool to characterize the quality of each study in four domains (Patient Selection; Index Test; Reference Standard; Flow and Timing)^18,19^. Two independent authors assessed the risk of bias, resolving between them any disagreement. To ascertain the quality of the review and evidence produced we followed the Prisma checklist^7,8^ and GRADE evaluation tool^40^, respectively (Table 3).

**Table 3:** Summary of findings.

Patients: Adult women with pathology proven pure DCIS Interventions: Magnetic resonance imaging Comparison: Pathology analyses	
Outcome: Accuracy in tumour size measurement	
Reported accuracy	Accuracy rates (weighted mean)
Accurate	54.74%
Overestimation	26.69%
Underestimation	18.57%
Mean size difference (mm)	Certainty of the evidence (GRADE)
Overall—3.85, CI 95% [−0.92;8.60]	⊕⊕⊕ο Moderate^a^
3 T MRI—3.47, CI 95% [0.02, 6.92]	⊕⊕⊕ο Moderate^a^
1.5 T MRI—7.18, CI 95% [3.34, 11.01]	⊕⊕⊕ο Moderate^a^
Acquisition time <0.3 s—1.97, CI 95% [−0.63, 4.56]	⊕⊕⊕ο Moderate^b^
Acquisition time >0.3 s - 3.03, CI 95% [0.25, 5.81]	⊕⊕⊕⊕ High
Temporal resolution <60 s: −1.89, CI 95% [−4.7, 0.9]	⊕⊕⊕ο Moderate^b^
Temporal resolution >60 s: −8,71, CI 95% [−13.8 −3.6]	⊕⊕⊕οο Low^a,b^
Slice thickness <1.5 mm: 3.53, CI 95% [1.63; 5.43]	⊕⊕⊕⊕ High
Slice thickness >1.5 mm: 6.94, CI 95% [0.04; 13.63]	⊕⊕οο Low^a,b^
Low risk of bias—2.46, CI 95% [0.57,4.36]	⊕⊕⊕ο Moderate^a^

GRADE: tool to assess the certainty or quality of evidence^33^. Classification according to the following levels:

⊕⊕⊕⊕⊕ High: a very good indication of the likely effect is provided.

⊕⊕⊕O Moderate: a good indication of the likely effect is provided.

⊕⊕OO Low: some indication of the likely effect is provided.

⊕OOO Very low: indication of the likely effect is provided is not provided.

^a^The certainty of evidence was downgraded in one level due to inconsistency (presence of moderate to considerable heterogeneity).

^b^The certainty of evidence was downgraded in one level due to low number of included studies/measured tumours.

## Supplementary information

Supplementary material

## Data Availability

Abstracted and imputed data used for the meta-analysis are presented in the supplementary information. All supplementary information is available at the npj Breast Cancer website. The complete version of abstracted data is available upon reasonable request to the corresponding author.
