# Supplementary material for: The accuracy of magnetic resonance imaging in predicting the size of pure ductal carcinoma in situ: a systematic review and meta-analysis
Source: NPJ Breast Cancer. 2022 Jun 29;8:77. doi: 10.1038/s41523-022-00441-x (PMC9243148; doi:10.1038/s41523-022-00441-x)
Supplement: Supplementary file 1 — Supplementary material [file 41523_2022_441_MOESM1_ESM.pdf]

Supplementary table 1: abstracted DCIS size measurements and correlations coefficients, identifying imputed values

papers	year	Number of DCIS	Pathology size	Pathology size SD	MRI size	MRI size SD	Correlation Coefficient
Song et. al	2020	50	25.0	18.0	25.8	15.5	0.620
Sanderink et. al	2020	46	21.8	17.5	19.2	10.5	0.710[1]
Baek et. al	2017	55	20.0	18.0	23.0	17.0	0.681[1]
Daniel et. al	2017	244	18.8	13.6	25.3	18.4	0.409
Brennan et. al	2017	16	42.3	19.0	58.3	34.6	0.419
Rahbar et. al	2015	19	14.1	13.2[1]	18.2	15.1[1]	0.660
Pickles et al.	2015	26	50.6	34.2	49.6	26.8	0.596[1]
Baur et. al	2013	58	39.1	24.0[1]	43.6	31.5[1]	0.740
Shin et. al	2012	88	30.5	20.1	31.7	21.3	0.513[1]
Marcotte-Bloch et. al	2011	32	25.6	20.0	28.1	20.8	0.831
Leung et. al	2010	9	18.0	11.1	27.4	21.2	0.912
Vanderwalde et. al	2010	9	28.3	28.8	6.4	8.8	0.800[1]
Onesti et. al	2008	16	18.2	4.9	31.1	4.4	0.892[1]
Kumar et. al	2006	45	32.4	12.8[1]	47.6	26.5[1]	0.676[1]
Menell et. al	2005	34	7.0	1.8[1]	20.0	11.7[1]	0.681[1]

1 Imputed values

Supplementary table 2: Table showing assessment of the risk of bias of the included studies, according to QUADAS-2.

	RISK OF BIAS				APPLICABILITY CONCERNS		
**Study**	PATIENT SELECTION	INDEX TEST	REFERENCE STANDARD	FLOW AND TIMING	PATIENT SELECTION	INDEX TEST	REFERENCE STANDARD
Menell 2005	?	☺	☺	☺	☺	☺	☺
Esserman 2006	☹	?	☺	☺	?	☺	?
Kumar 2006	☹	☹	?	?	?	☹	?
Onesti 2008	☺	☺	?	☺	☺	?	☺
Marcotte-Bloch 2009	☺	☺	☺	☺	☺	☺	☺
Leung 2010	?	?	?	☺	☺	☺	☺
Allen 2010	☺	☺	☺	☺	☺	☺	?
Vanderwalde 2010	☺	☹	?	?	☺	☺	☺
Shin 2012	?	☺	☺	☺	☺	☺	☺
Baur 2013	☺	☺	☺	☺	☺	☺	☺
Mun 2013	?	☺	☺	☺	☺	☺	☺
Gruber 2013	?	☺	☺	☺	☺	☺	☺
Pickles 2015	☺	☺	☺	☺	☺	☺	☺
Rahbar 2015	☺	☺	☺	☺	☺	☺	☺
Rominger 2015	☺	☺	☺	☺	☺	☺	☺
Baek 2017	☺	☺	☺	☺	☺	☺	☺
Daniel 2017	?	☺	?	?	☺	☺	☺
Brennan 2017	☺	☺	☺	?	?	☺	☺
Preibch 2019	?	☺	☺	☺	☺	☺	☺
Song 2020	☺	☺	☺	☺	☺	☺	☺
Sanderink 2020	☹	☺	☺	☺	☺	☺	☺
Shiraishi 2020	☺	☺	☺	☺	☺	☺	☺

☺ Low Risk ☹ High Risk ? Unclear Risk

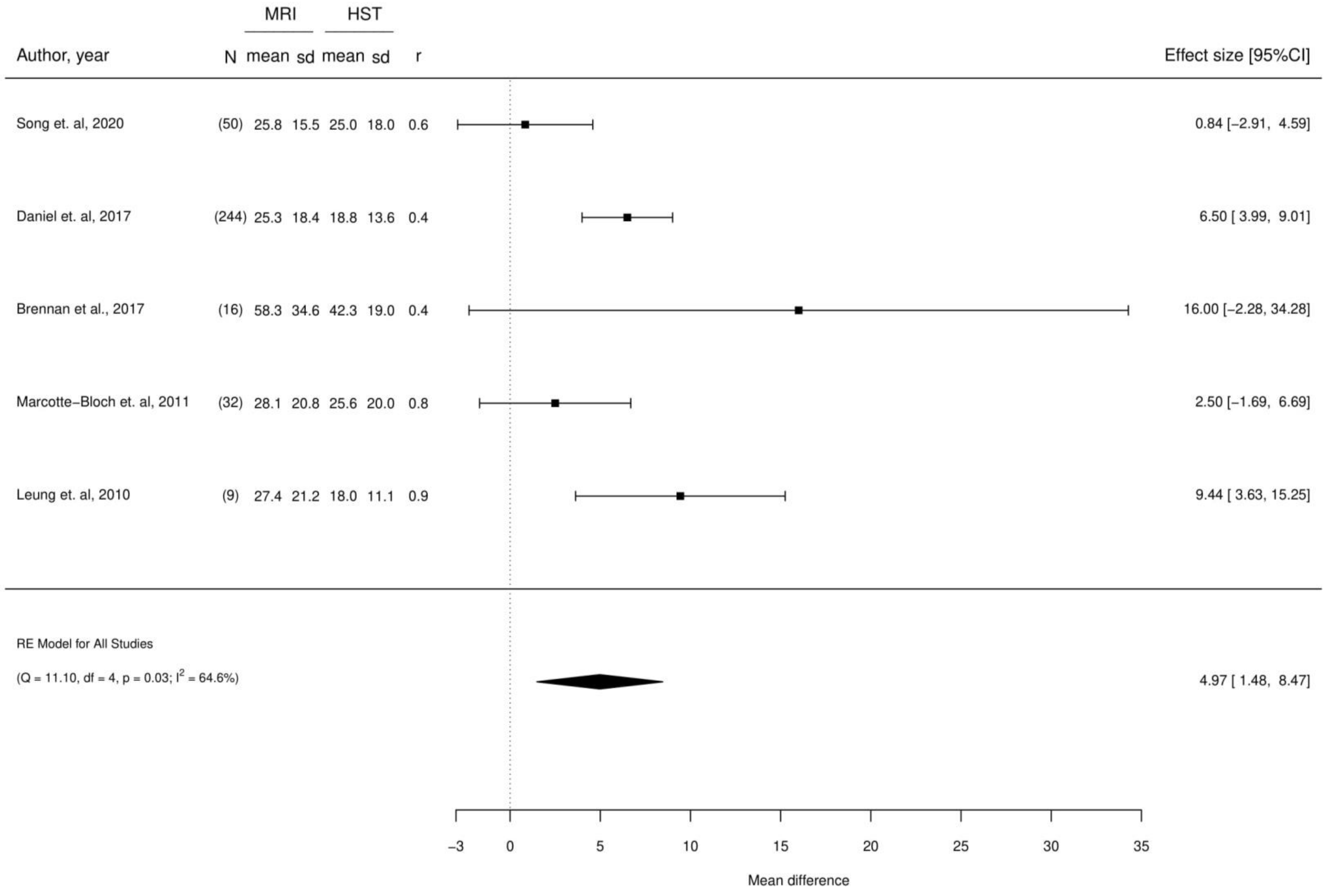

Supplementary figure 1: Pooled results of the mean size difference of paired measurements of DCIS with MRI and pathology, without using imputation method. In the absence of imputation, only 5 articles are eligible for meta-analysis.

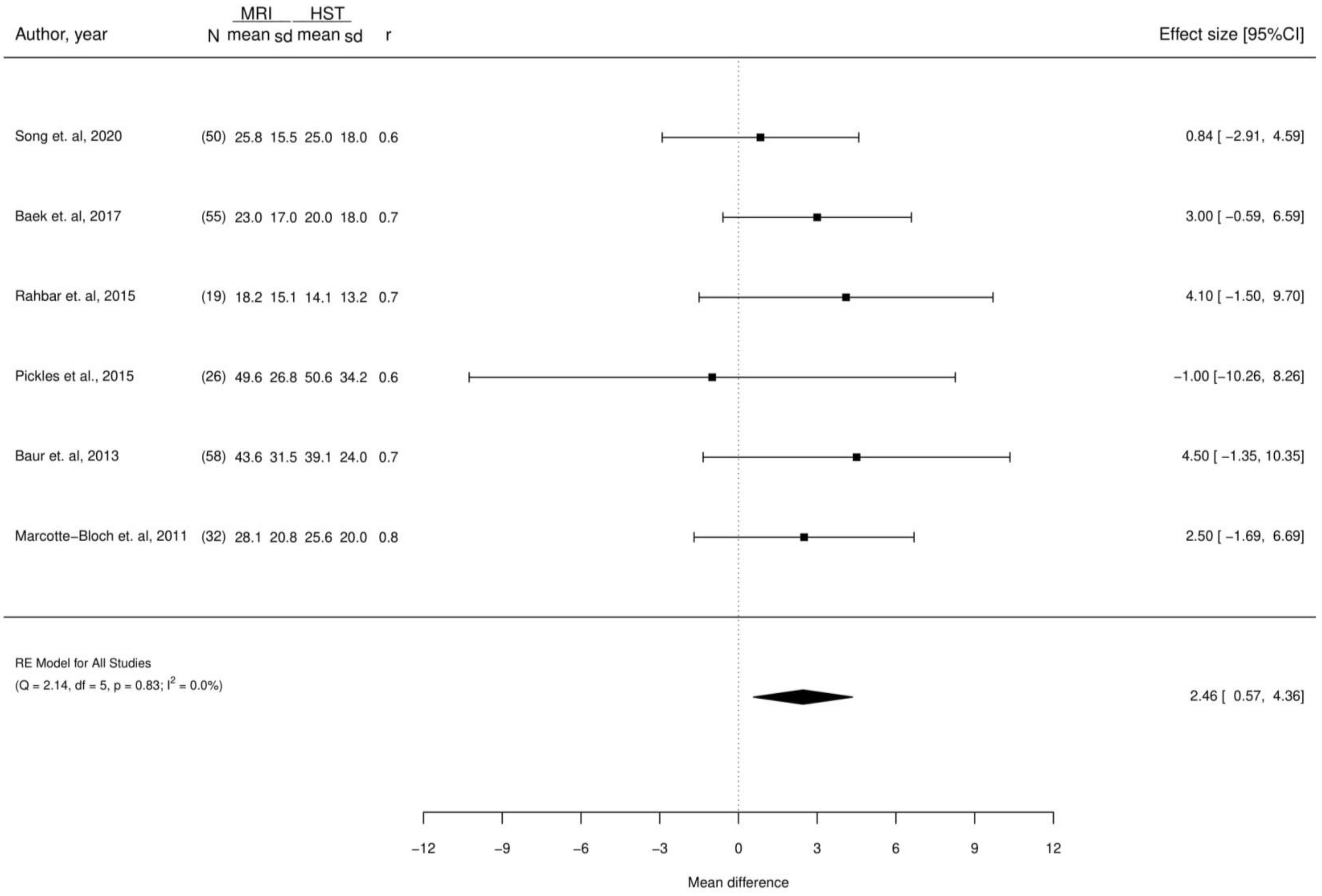

Supplementary figure 2: Pooled results of the mean size difference of paired measurements of DCIS with MRI and pathology, in studies evaluated as having a low risk of bias.

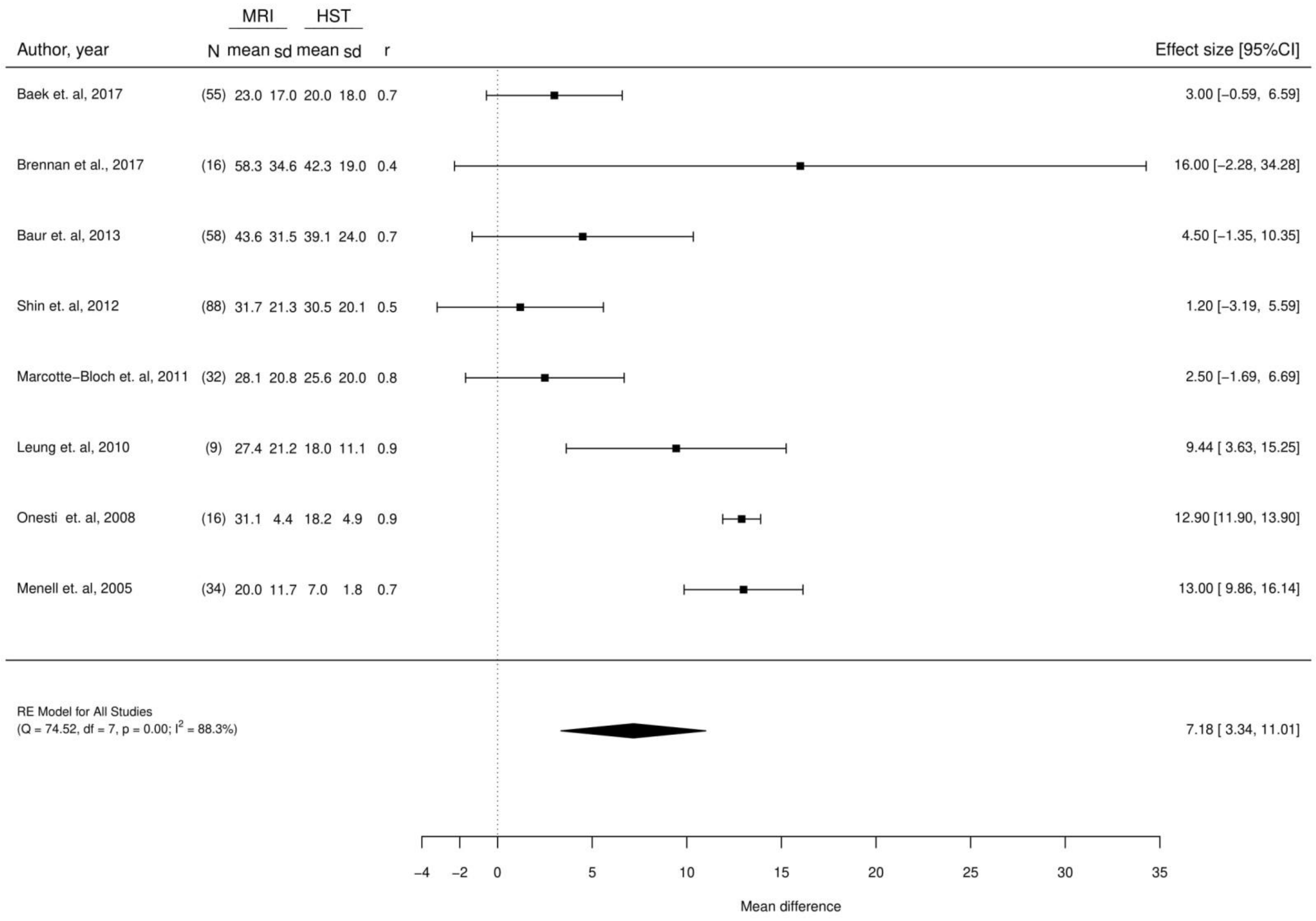

Supplementary figure 3: Pooled results of the mean size difference of paired measurements of DCIS with MRI and pathology, in studies using 1.5T MRI.

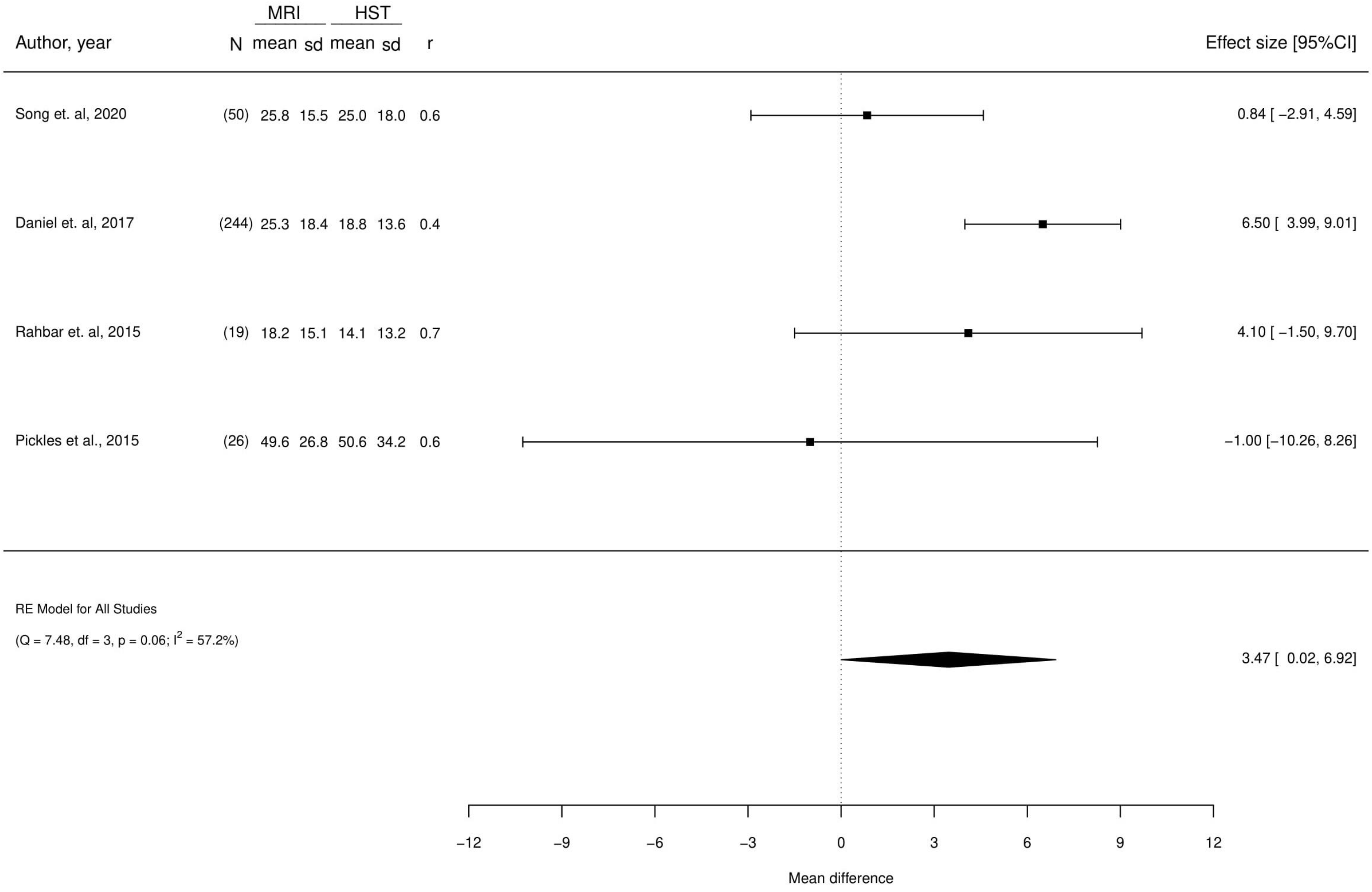

Supplementary figure 4: Pooled results of the mean size difference of paired measurements of DCIS with MRI and pathology, in studies using 3T MRI.

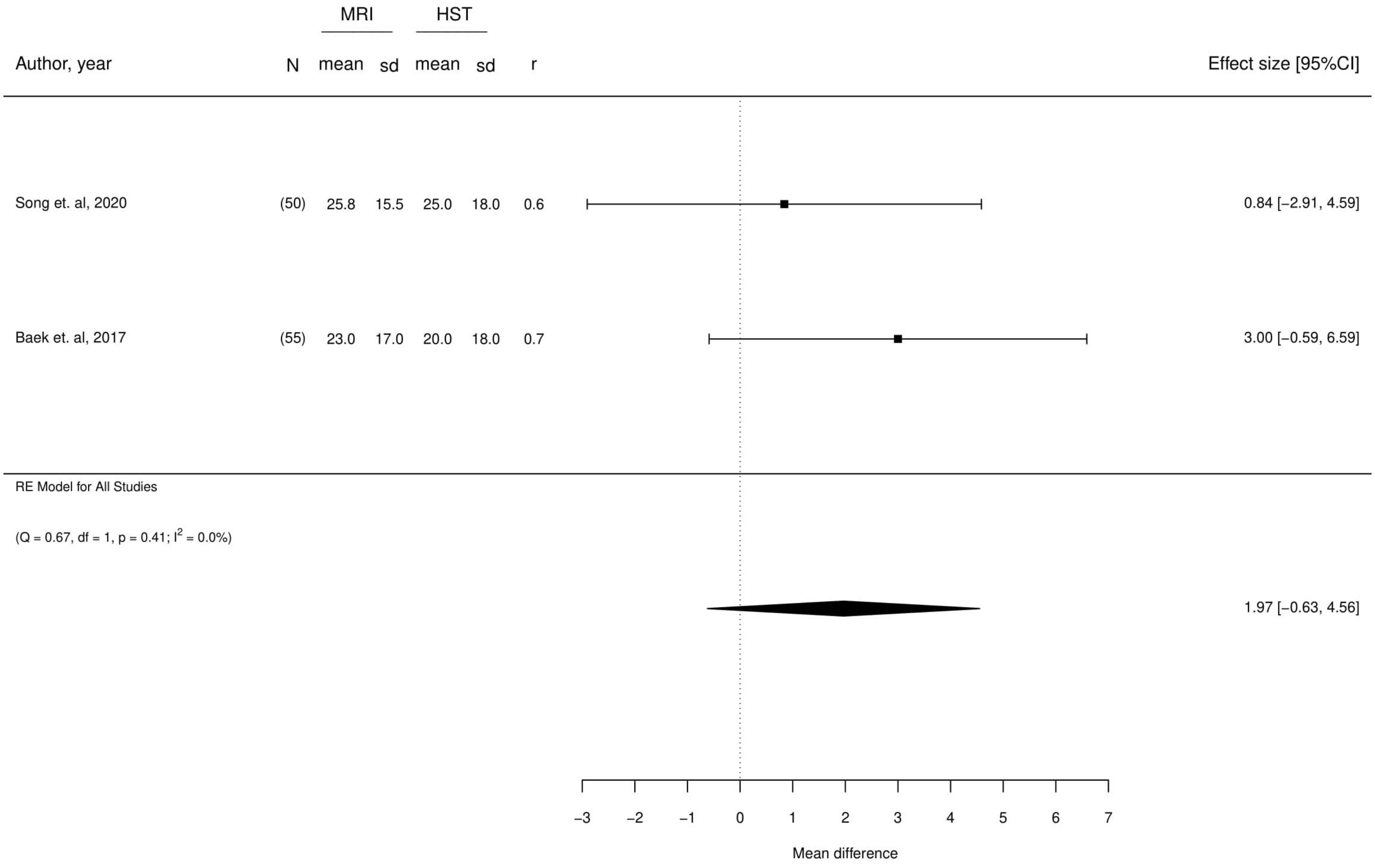

Supplementary figure 5: Pooled results of the mean size difference of paired measurements of DCIS with MRI and pathology, in studies with DCE-MRI scan time below 0.3s.

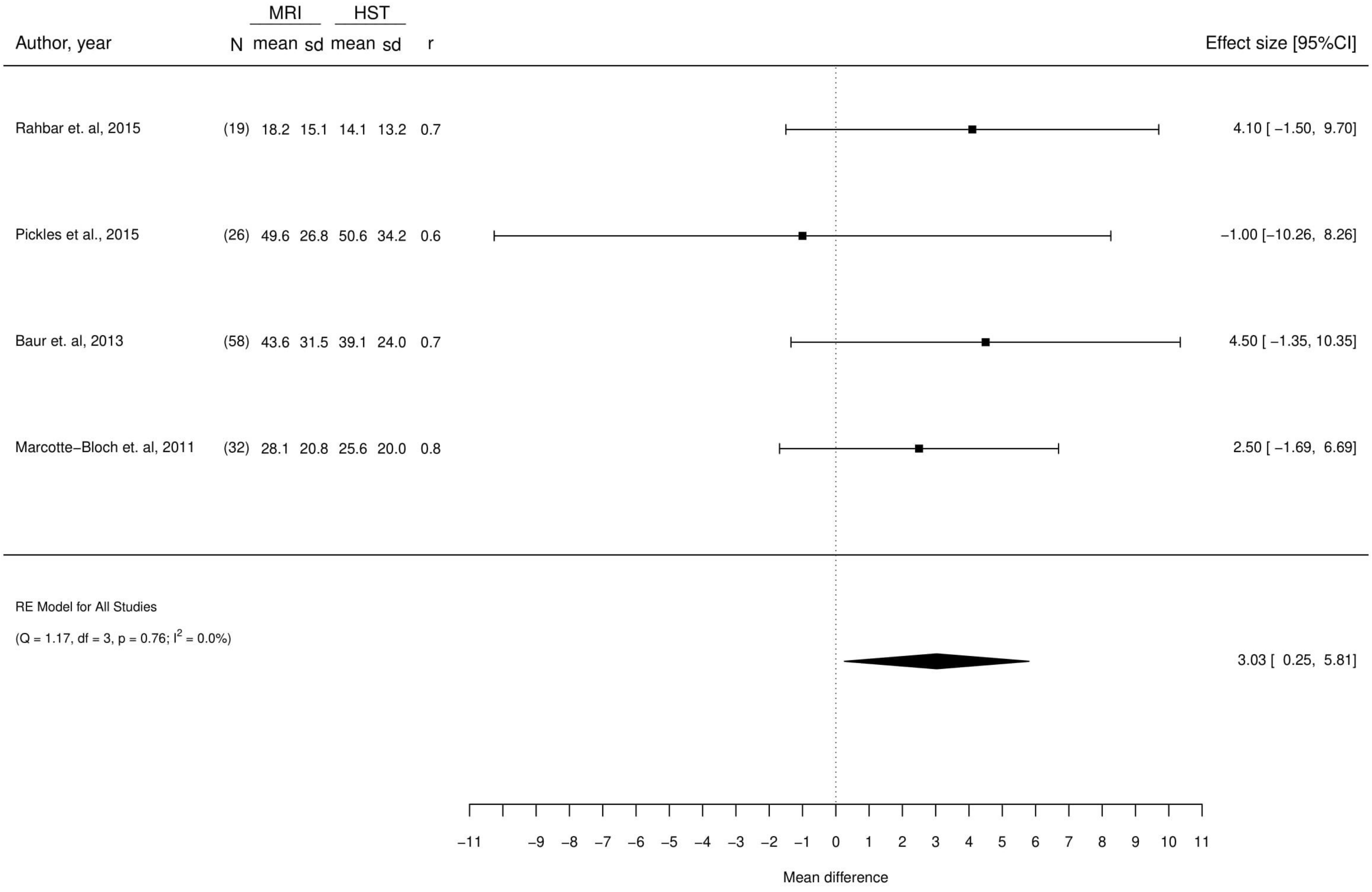

Supplementary figure 6: Pooled results of the mean size difference of paired measurements of DCIS with MRI and pathology, in studies with DCE-MRI scan time above 0.3s.

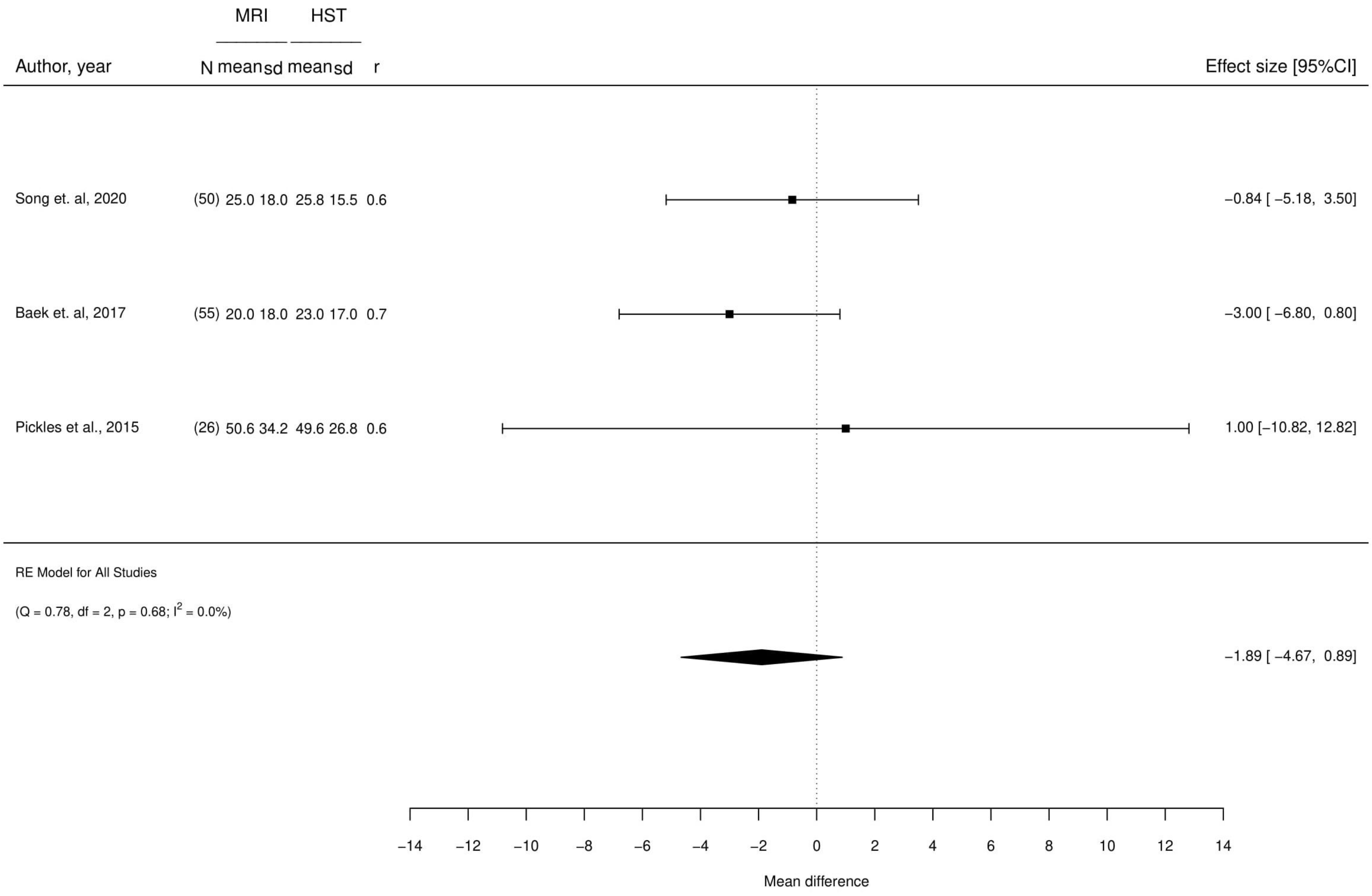

Supplementary figure 7: Pooled results of the mean size difference of paired measurements of DCIS with MRI and pathology, in studies with MRI temporal resolution of 60s or less.

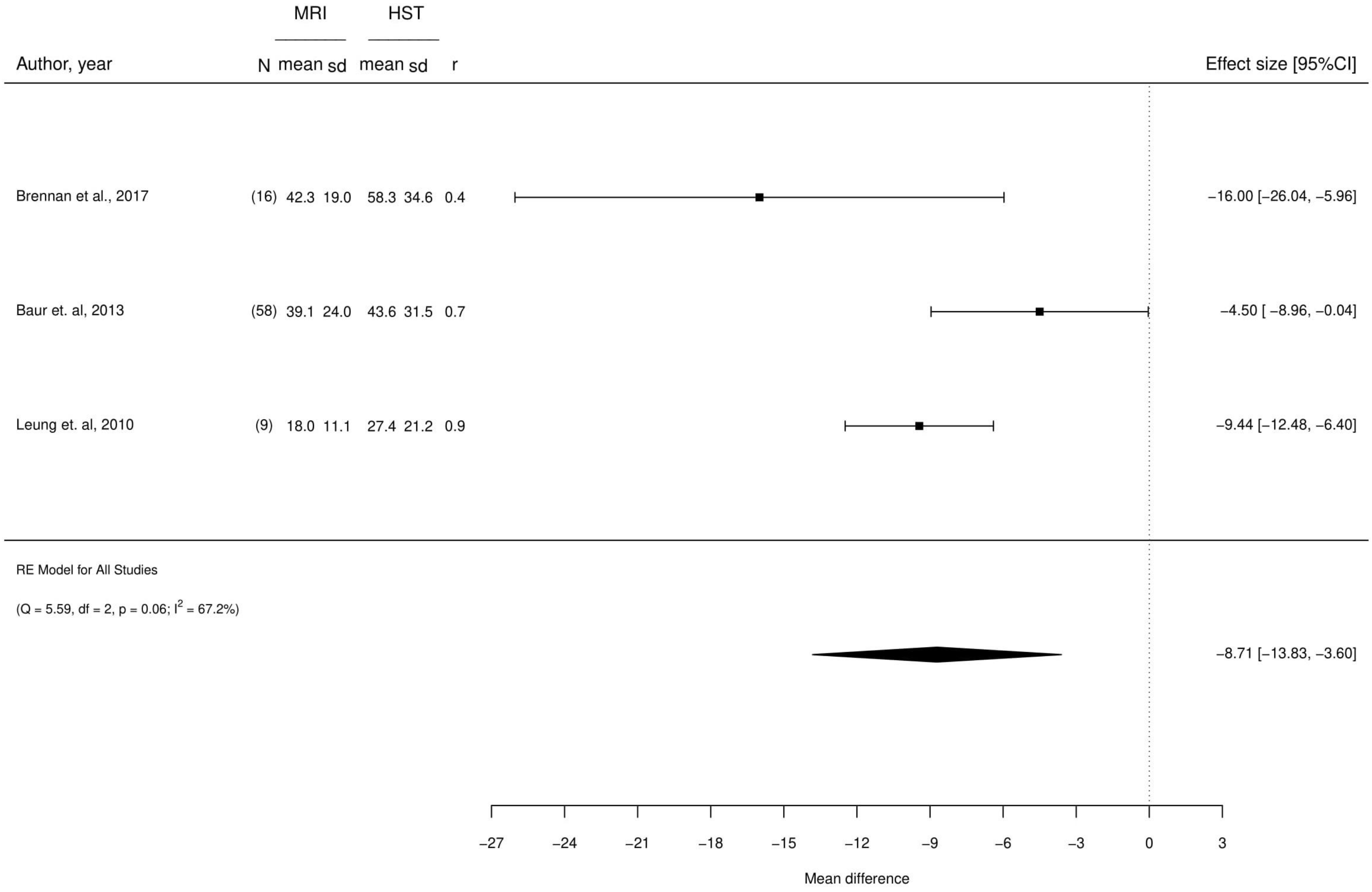

Supplementary figure 8: Pooled results of the mean size difference of paired measurements of DCIS with MRI and pathology, in studies with MRI temporal resolution greater than 60s.

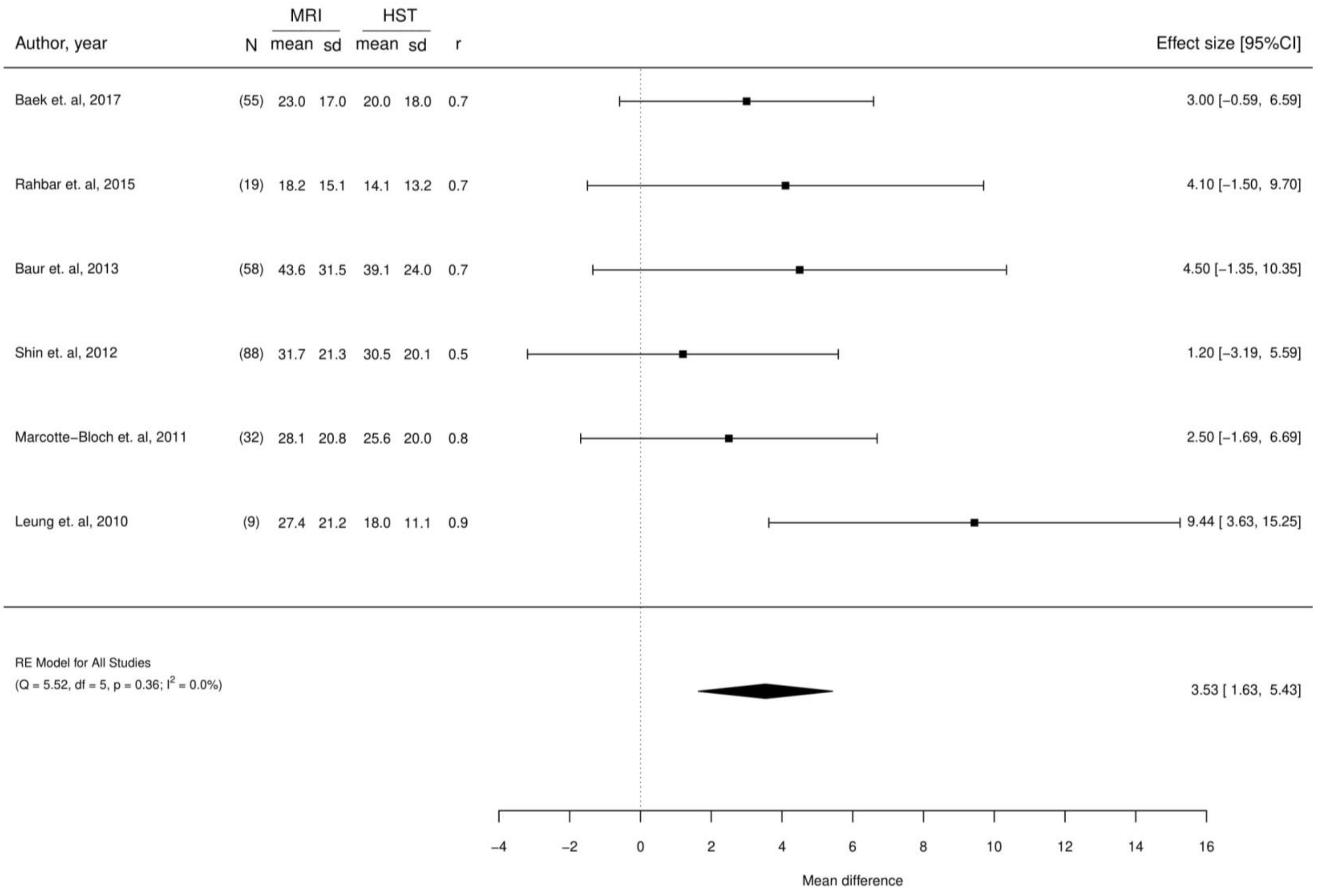

Supplementary figure 9: Pooled results of the mean size difference of paired measurements of DCIS with MRI and pathology, in studies with MRI slice thickness of 1.5mm or less.

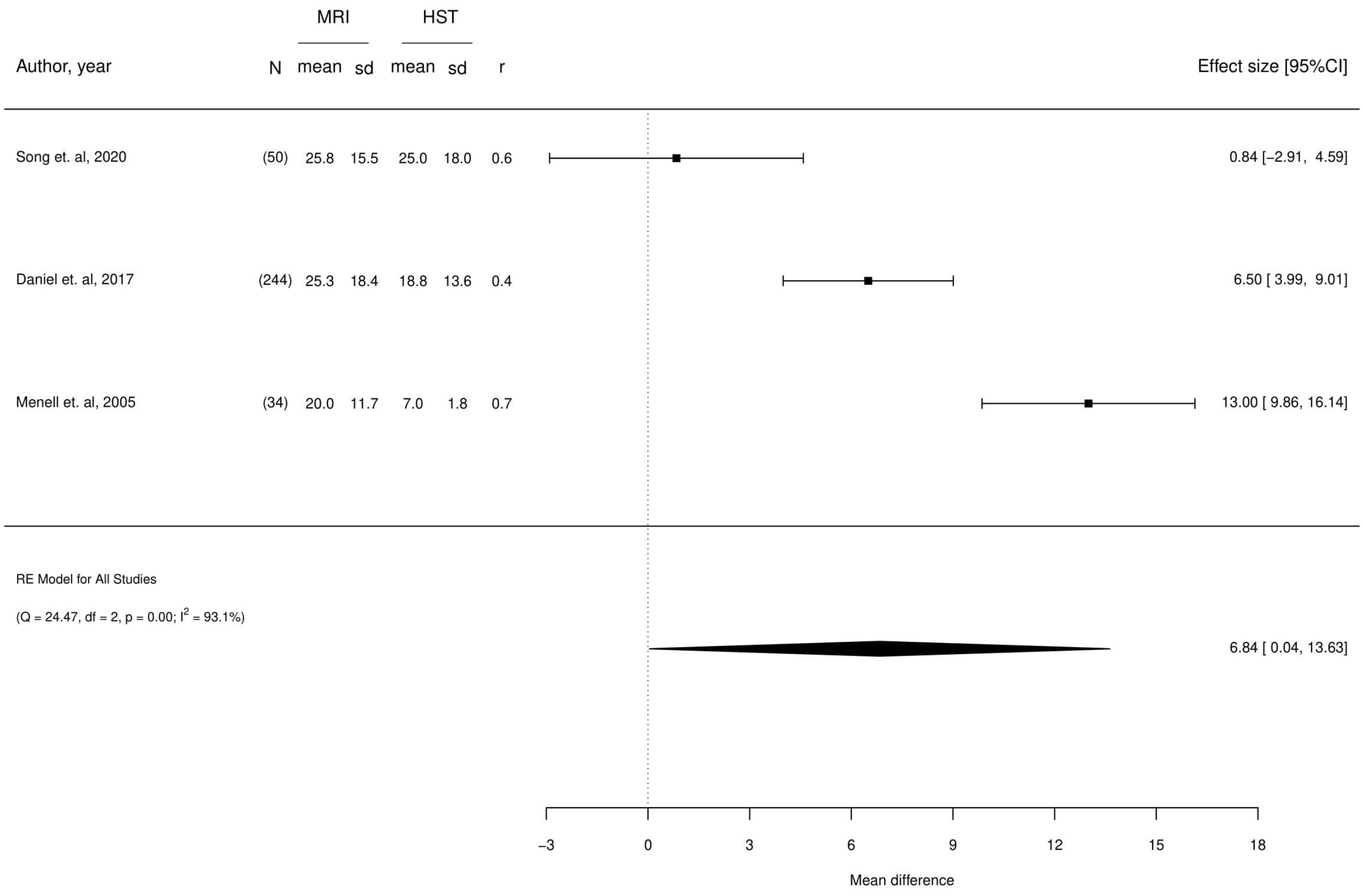

Supplementary figure 10: Pooled results of the mean size difference of paired measurements of DCIS with MRI and pathology, in studies with MRI slice thickness above 1.5mm.

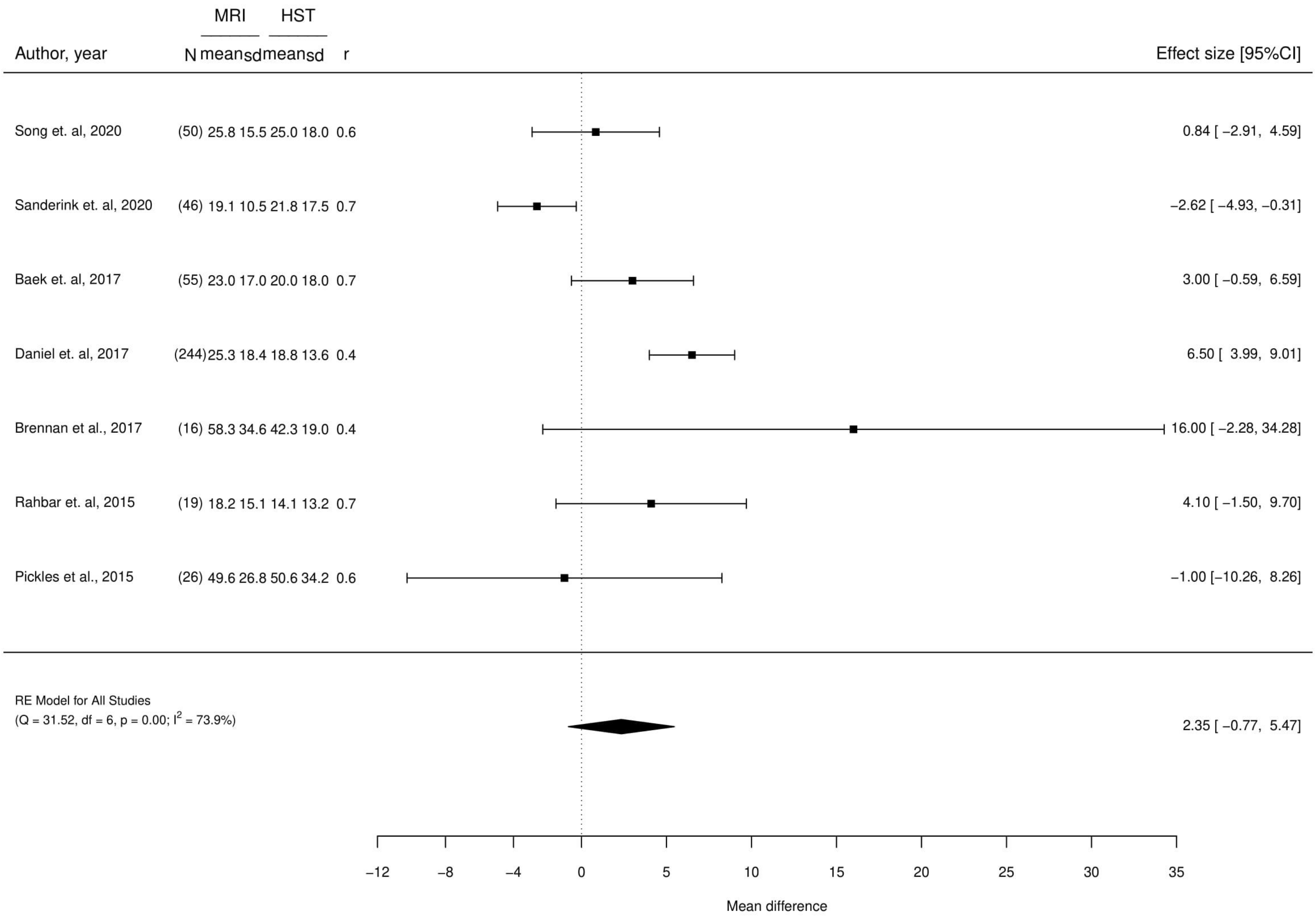

Supplementary figure 11: Pooled results of the mean size difference of paired measurements of DCIS with MRI and pathology, in studies published in the last five years.
